# The Nature and Treatment of Diseases of the Ear

**Published:** 1838-07-01

**Authors:** 


### The Nature and Treatment of Diseases of the Ear. By Dr. *William Kramer*. Translated from the German, with the latest Improvements of the Author since the last German Edition, by *James Risdon Bennett*, M.D., &c. London, Longman, Orme, & Co. Pp. 306.

One of the uniform results of civilization, with its attendant increase of wealth and population, is the minute division of labour, and the breaking up into the smallest fractions the pursuit of every art and science. Thus medicine, which as a science grasps all that relates to the human organization and its injuries, was first broken into two grand divisions—medicine and surgery. These in the present day are again subdivided into almost as many parts as there are diseases or evils to which "the flesh is heir." It is worthy of remark also that these minute subdivisions obtain much more in surgery than in medicine, and increase in proportion as we descend to those branches in which manual dexterity, rather than mind, is most called into play. In the higher paths of the art, although a surgeon's reputation may be great and apparently referable to a particular class of operations, yet it never happens that he does not also practise every other, and generally with equal or distinguished talent and success. As we gradually descend, however, to oculists, aurists, dentists, &c., that general professional ability disappears—the *science* merges into the *art*, until we come to the dentist and aurist, who are nothing more than dentists or aurists—clever workmen in a mechanical art, with only the local science appertaining to their branch of occupation.

This order of things, while it produces some beneficial results, presents many disadvantages. If, with the general knowledge and scientific attainments required for the efficient practice of medicine in its comprehensive sense, the attention is *subsequently* devoted to any one fraction or branch of the science exclusively, there is little doubt but that such concentration upon one point is calculated to effect the most important and valuable results. The greatest manual dexterity is only one of the most obvious. Tact and power of discrimination, in all that relates to the point in question, increased knowledge of the functions, nature, and structure of the particular organ or part so constantly and intently studied, are still more important, tending to increase the general stock of pathological and physiological knowledge, and, by improving a part, lead to the gradual perfection of the whole art or science.

On the other hand, the very nature of such study, limited and concentrated as it must be, tends injuriously to contract the views, by drawing the mind from the general and more comprehensive contemplation of the whole frame and system, with its almost infinite collateral relations, so essential to a due appreciation of the disease of any part. This tendency will act more or less even upon enlarged minds; and where previous study has not given habit of comprehensive reasoning, the inevitable result must be the practice of many important branches of surgery as mere mechanical arts. When we reflect that the branches which most habitually fall into such hands are those which relate to the senses, and upon the perfection of which the comfort and usefulness of a life depend, it is worthy of serious consideration to which side the balance inclines—to injury or benefit. It leads also to a practical question of the highest importance—the degree of protection which it may be desirable that every legislature should afford to the community. Whether it be not incumbent upon law-makers, in all nations, to require proof of general scientific attainments fitting the possessor to practise *every* branch of the profession, before he be permitted to devote himself to any *one*. In our opinion this ought to be a *sine quâ non*, a fundamental principle in all legislation on the subject. The divisions in practice, by which individuals limit themselves to a peculiar province, would then spring legitimately from the wants and interests of society—be regulated and controlled by them, and would not, as at present, be determined by the interest of the individual alone, often in opposition to that of the community. The public and the profession would alike benefit—there would be fewer of these subdivisions—those which existed would tend to improve the art, and when we gave our ear to an aurist, we should be protected from the chance of deriving mischief instead of benefit from the operation—an accident which, if we may believe these gentlemen themselves, has not seldom happened.

These reflections have arisen naturally from the perusal of the work before us, which at once illustrates some of the advantages and the disadvantages described. Here we find overdrawn distinctions without important differences—little things made great by the undue importance lent to them, and a portly volume instead of a small octavo. We find in this work, however, ample evidence that both the original writer and the translator possess the scientific attainments already described as essential to the proper study, practice, or improvement of any branch, and the joint result of their labours will be found to form a step of great importance in the advancement of our knowledge of the nature and treatment of this class of diseases.

The author says in his preface, "that the work before us is no longer a fragment—a work on the important Chronic Forms of Diseases of the Ear; but an exposition as complete as possible of Systematic Acoustic Medicine." It is divided into two parts. The first commences with a critical survey of the literature extant on the subject, and an inquiry into the general pathology and therapeutics of diseases of the ear, pointing out the errors, ancient and modern. The second part gives a comprehensive system of all diseases of the ear, arranged according to the structural alterations of the parts of the organ affected—illustrated by various cases, and concluding with some observations on ear-trumpets and deaf-dumbness.

The history of acoustic medicine is reduced to a wonderfully small compass; from the first dawn of medical science, in the time of Hippocrates, down to our own times, little or nothing was known of the diseases of the ear. The author says—

"For more than a thousand years, these crude empirical principles of Galen maintained undiminished and entire authority. The inestimable anatomical discoveries respecting the ear, that were made towards the end of the fifteenth and in the first half of the sixteenth century, by Achillini, Berengar, Vesalius, Ingrassius, Eustachius, Fallopius, Casserius, and others, had not the least influence on the pathological and therapeutical views of the practitioners of that period." 6.

The whole practice seemed to consist of the application of aromatics and violent stimulants to the external organ. Mercurialis, who wrote in 1591, and whose treatise obtained for a time great celebrity, " recommends earnestly" the practice of holding a child up by the heels and shaking him, to dislodge a foreign body from the ear—and tying a man on a plank with the ear affected to the board, that he might be well and conveniently shaken! We need not therefore go further as regards the 16th century.

Fabricius Hildanus, who published in 1646, was the first to investigate these diseases, but seemed to direct his attention to the external meatus only, and its morbid states. He invented the first speculum auris. Bonet, thirty years later, has the enviable distinction of giving examples of how " dissections of the internal ear should *not* be made." Du Verney, who published towards the end of the 17th century, was the first who contributed essentially to the improvement of our knowledge of the ear—for he alone began by a minute study of the anatomy, structure, and function of the organ. This was a great step, and although he may have fallen into some errors in his pathological deductions, we by no means agree with Dr. Kramer in the blame he would attach to him, even supposing it to be correct that " his pathological views seem altogether confused." After floundering in the dark some twenty centuries, surely no small degree of praise is due to him who first finds the right road, and even advances considerably in the path. It is too much the custom for modern writers to arrogate to themselves exclusively the whole merit of the present state of any science they may have improved, wilfully closing their eyes to the fact, otherwise sufficiently obvious—that they have merely made a few steps in advance of a great many which had been made before them by those who preceded, and under much less advantageous circumstances. Thus Dr. Kramer's labours have been founded upon the important series of anatomical discoveries which had their commencement in the 16th century:—upon the pathological labours of Itard, Deleau, Abercrombie, &c.

It is a curious circumstance, that the discovery of the Eustachian tube had taken place nearly two hundred years before it was turned to any account, and then, more strange still, by a post-master of the name of Guyot, who injected his own to relieve deafness. This, by far the most important step made in the investigation and treatment of diseases of the ear, seemed never to have entered into the contemplation of the aurists. Self-interest gives a wonderful keenness to all the faculties, and the post-master, who evidently must have obtained a considerable degree of anatomical knowledge, was enabled to seize upon a relation between the anatomy and the diseases of the ear, and devise a method of treatment which for two hundred years had entirely escaped those whose peculiar study it was.

" Notwithstanding the great imperfection of Guyot's method; that is to say, notwithstanding the introduction of the instrument through the mouth, a plan now altogether abandoned, and replaced by the more correct method of introducing it through the nasal fossa, Guyot's discovery still forms an epoch in the history of acoustic medicine; for it has afforded a fixed, and indeed a most sure basis, for understanding and treating diseases of the middle and internal ear." 10.

After enumerating many authors of the 18th century, the author comes to the conclusion that—

" With all these defects in the best literary productions of the time, the treatment of acute diseases of the ear was tolerably successful in ordinary practice; the more evident general therapeutical indications were adopted with success, patients were treated by antiphlogistic remedies, both general and topical. But, towards the close of the eighteenth, and at the beginning of the nineteenth century, the information of physicians did not extend beyond acute diseases; of which Lentin's unsuccessful attempt to advance to the knowledge of chronic diseases of the ear, affords the best proof." 11.

After dealing in the most summary manner with authors of the 19th century, German, Dutch and French, the English are reviewed, and certainly do not pass muster; of Curtis—he says

"From whom, as the head of a large institution for the treatment of diseases of the ear, verily better performances might have been expected. Curtis treats every discharge from the ear, exclusively, and in a summary way, by means of astringents; obstruction of the Eustachian tube with emetics, and perforation of the membrana tympani; whilst, in spite of all the entreaties of Saissy, he has never once practised catheterism of the Eustachian tube on the living subject. He makes tinnitus the chief symptom of nervous deafness, which he treats with purgatives, especially calomel, as long as the strength of the patient holds out. In all doubtful cases his chief attention is directed merely to ascertain whether the liquor Cotunnii be partially or totally deficient!! or, whether hardened wax exist in the meatus."

"In the otitis of children he sticks opium into the affected ear, &c.; so that throughout all his writings, nothing but the most crude empiricism is to be met with; and yet among his compatriots, as well as abroad, Curtis generally possesses the reputation of being a distinguished aurist." 17.

At last the writings of Itard and Deleau appeared, and seem to have formed an era in acoustic medicine.

"Itard has unquestionably the merit of having treated diseases of the ear more comprehensively, more methodically, and with more critical acumen, than had ever been done before." 21.

Deleau's industry only extended to the diagnosis and treatment of diseases of the middle ear.

So much for the "*critical literary review*" which may be summed up in one sentence. It is only of very late years that any valuable knowledge has been obtained of the diseases of the ear, and their treatment. Itard and Deleau, by their works and practice, threw considerable light on the subject, Du Verney, chiefly by his anatomical labours, having led the way. The condemnation of all writers is so sweeping that the literary history resolves itself very nearly into a history of dates.

The object of the whole work is thus defined by the author.

"It has also been my endeavour to arrange diseases of the ear in a more natural manner than has hitherto been done; to refer them to definite organic alterations of the constituent parts of the ear; to avoid all hypothetical and speculative assumptions; and to establish the diagnosis of each form of disease by *the exposition of objective symptoms, independent of the ever doubtful accounts of the patients, and on this sure basis to establish a plan of treatment as simple and certain as possible.*" 23.

The anatomy of the ear we are told has reached an "almost unexampled state of perfection"—while the efforts to discover and establish the physiological importance of the particular constituent parts of the ear have been vain. This statement seems at variance with the general tone of the work, which leads to the conclusion, that acoustic medicine has arrived at a considerable degree of excellence—for it is an axiom which hardly admits of dispute, that the pathology of an organ cannot be thoroughly understood until its physiology is well and accurately known.

It appears also, that whatever be the disorder, whether organic or functional—acute or chronic—in the external, middle or internal ear, there is one single symptom indicative of all without exception, and even " whether these diseases have their seat originally and exclusively in the ear, or whether the latter suffer sympathetically from the affection of some other organ." And here seems to lie the chief difficulty, for it not only is a universal symptom, but often the one only—defective hearing.

On the progress of these affections, Dr. Kramer observes, that diseases of the ear are disposed to run a chronic course unattended by fever—they are but very rarely truly acute.

" On an average, not more than two out of a hundred patients labouring under diseases of the ear will be found, whose disease assumes a really acute character. The rest all labour under forms of disease, which have from the first been of a chronic character, or such as are attended originally by a slight inflammatory excitement, which, however, soon merges into a morbid secretion from the affected parts, and assumes a purely chronic form. The reason of this peculiarity depends on the solid structure of the ear, composed of bone, cartilage, and membranes firmly stretched over these, and which is but sparingly supplied with cellular tissue, and with an equally small proportion of blood-vessels." 31.

" Yet it is of the utmost importance to know, that diseases of the ear admit of a *very certain diagnosis,* (not indeed according to the usual old established mode,) that in general they run a very chronic course, and that under the influence of these two circumstances, *they are almost all curable,* if the treatment *of them be only undertaken in proper time, and with the proper remedies.* But this right moment of time is, to the great prejudice of the patients, (partly from their own faults, and partly from that of ill-informed practitioners,) almost always neglected, and thus the disease is, artificially and quite contrary to its nature, rendered incurable." 35.

" *The degree of the dulness of hearing, the age of the patient, and even the length of time that the disease has existed,* of themselves afford absolutely no prognostic data. But the *degree of organic change* and of *functional disturbance* to which the disease has attained, are indeed of the greatest importance." 36.

" Organic diseases of the ear are in general both cured and prevented from recurring after being successfully cured, with more certainty than functional diseases of the same organ, the causes of which are more difficult of recognition, and their progressive influence, and their renewal, much less under our control than is the case with organic diseases."

" My opinion regarding the curability of diseases of the ear in general, (their individual curability will be considered in another place,) is founded on the results of 300 cases, as they have been recorded in my journal according to the order of time in which they occurred, without any selection, and after having been investigated in the most careful and complete manner." 37.

" Of these 300 patients, 104 were found to be quite incurable, incapable of being at all relieved, and with the treatment of which, therefore, I took no trouble; the proportion of these, therefore, is one to three. On the other hand, 188 were either completely cured or relieved by the treatment, whilst only eight of those who were actually put under treatment, were obliged to be left unrelieved, in spite of all the pains and care bestowed." 38.

This we think by no means a favourable statement of the art. Out of 300, one third are altogether hopeless; and even of the other two-thirds, of which the majority are classed as "cured or *relieved,*" we are not told how many are *cured,* and if only relieved, by Dr. Kramer's own opinion, they must be supposed to relapse.

The author doubts whether lues acts in any specific mode in producing diseases of the ear, stating, that he has never met with any confirmative observations, either in his own practice or in that of others. In more than one case we have traced a direct connexion, where lues affecting the head has undoubtedly produced disease in the ear—the throat has however been more or less affected. We are not prepared, therefore, to maintain its *specific* character.

The remedies which have been, or now are most in vogue, are elaborately examined, after being divided into remedies of local action, and remedies of general action. Of these it may be said as of the authors, they are nearly universally rejected by Dr. Kramer as more or less injurious or inefficient. And indeed remedies can only be rendered of value by the knowledge which dictates their use; if that is defective, it must be a very happy chance which allows their use to be productive of benefit.

Thus of the local remedies most resorted to, he says to electricity can only be attributed one case of cure. Saissy limits its use to incomplete paralysis of the auditory nerve. Itard and Deleau, Dr. K's two great authorities, says it has no beneficial influence. "Electricity and Galvanism are utterly useless, and not free from danger, by their debilitating action on the auditory nerve." And in support of this conclusion there is a most formidable mass of evidence brought forward.

Moxa and actual cautery in any point of view, are held to be too powerful for the auditory nerve. Blisters and tartar-emetic ointment applied behind the ear, are described as often useless, sometimes injurious, and only indicated in topical circumscribed inflammatory affections of the meatus and of the membrani tympani.

Issues have never appeared to exert any essential beneficial influence on the diseased ear.

"*Of Setons*—in the neck the same holds good as of issues; there does not exist a single case in which these have been made use of with undoubted advantage in diseases of the ear, and in which, at the same time, the affected organ was carefully examined, where the same result would not have been obtained by milder and more certain methods; whilst all those patients that I have seen who had worn setons, have unanimously described their influence on the aural disease as injurious." 57.

Of Douches, he says, there is no kind of disease of the ear that affords a rational indication for douches to the external meatus, whether of water or of vapour. Drops and injections, especially those of an acrid, spirituous, irritating class, are all highly injurious. Warm fomentations, injections of warm milk, the vapour of elder and camomile-flowers, &c., are mere playthings, "by which the patient is amused" (?) but mild as they are, if used very hot, capable of inflicting serious injury.

*Leeches.*—"Topical bleeding is only called for in acute inflammatory affections of the ear, and then indeed in the most urgent manner."

Remedies of general action the author defines as those which, by modifying the vital action, and altering the degree of power throughout the whole system, are intended to re-act beneficially on the affection of the organ of hearing, and he enters into an enquiry of the efficacy of Russia vapour-baths, salt-water baths—emetics, purgatives, general bleeding—salivation, and arnica flowers. And speaking of them all collectively, he says, with very few exceptions, it is in vain to expect from them any beneficial re-action. And, in conclusion, adds, by far the greatest number of diseases of the ear, are of a simple nature and not accompanied by general diseases, which stand in any intimate connexion with the aural affection.

The table with which this critical review of authors and remedies concludes is both interesting and instructive. It shews (as far as the small number, three hundred, will permit) the frequency and degree of curability of different affections under Dr. Kramer's treatment.

The second and by far the most valuable part of the work treats of the diseases of the ear, with Dr. Kramer's views of their nature, seat, and appropriate treatment. They are divided into three classes:—Diseases of the external; middle; and internal ear.

The first belongs especially to the periods of childhood and youth, and those described as relating to the auricle are not peculiar to it alone—such as erysipelas, furuncula, &c. With reference to diseases of the meatus externus, we find that

"Deviations in the quantity and quality of the cerumen are generally unimportant attendants on other diseases of the ear, but very seldom exist independently, and then exert no important influence on the function of the organ. Diseases of the external meatus fall also under the class of inflammatory diseases; though they very seldom assume an acute, but almost always a chronic form; which form they very readily pass into, even when at their onset they are acute. As the auditory canal is especially active in childhood and youth as a secretory organ, but, as with advancing age, the supply of fluids to this part gradually diminishes, the diseases to which it is subject are of necessity especially frequent in childhood and youth; whilst manhood and old age are particularly subject to diseases of the middle and internal ear." 95.

After a startling enumeration of diseases of this not very complicated part of the organ of hearing, we come at last to the gratifying conclusion that "all diseases of the auditory passage depend on inflammation of its organic constituent parts." Now as these organic constituent parts are not more various than in nearly all other parts of the human body, we cannot see any necessity for elaborate classification, and holding all unnecessary complication to be a great evil, we are inclined to think, as far as we can judge from our own knowledge, enlightened too by a careful perusal of Dr. Kramer's labours, that aurists are addicted to many nice distinctions without a difference, and leading to no good practical result. In the hundred pages devoted to the diseases of the meatus externus, we see room for at most but two classes. Inflammation of the canal—and inflammation of the membrani tympani, and we are further led to believe that the one is little likely to exist to any extent without the other, and consequently that even this division is of little practical importance. The treatment for both being the same, or varying only in proportion to the degree of inflammation.

We shall not follow our author therefore into the recondite labyrinths and nice distinctions of erysipelatous inflammation of the meatus—inflammation of the glandular structure—of the cellular tissue—of the periosteum of the meatus, &c. &c.—distinctions, we shrewdly suspect, much easier to describe on paper than to establish in practice, setting aside their inutility. This refining tendency is characteristic of our German colaborators, at one time leading them into the unfathomable mysticisms of Kaut, at another, when combined with less intellect, into the infinitesimal absurdities of homœopathy.

Nothing can be more simple, we had well nigh said uniform, than the treatment adopted by Dr. Kramer in these variously described diseases—treatment, which we collect, rather from the cases furnished in great profusion, than from his descriptions, which are by no means clear, definite, or easily seized and classed. Cleanliness, effected by frequent syringing of warm water; when the inflammation leads to excrescences or sprouting granulations, pressure by slips of dried sponges, and a weak injection of the acetate of lead in solution, are recommended—if polypi exist of the pedunculated class, they are to be cut away and the acetate of lead applied to the roots, or lunar caustic—the last remnant of the root, however, is usually extremely sensitive to the action of the caustic, and success very rarely follows. The solution of the acetate of lead increased to gr. x. to ℥j. of water, sometimes acts better than the caustic, and after it has failed. Cases of polypi, which have a broad base, are considered well nigh hopeless. As little can be done for the elevations of the glandular structure occurring in the vicinity of the glandular growth, they must as yet be considered irremediable. " If the swellings in the meatus externus be spongy, broken up, and vesicular, no other means than the sponge compress is required, small smooth slips of which are introduced, and allowed to remain for twenty-four hours. In these, as in other elevations of the glandular integument, all emollient, mild, oleo-mucous remedies, fomentations, Russian vapour-baths, and the like, are injurious; they only increase the congestion of the morbid parts, and augment the secretion and relaxation. If the glandulous integument be swelled without being broken up, " there is nothing more efficacious than a solution of acetate of lead (gr. j. — x. to the ℥j. of water) dropped into the ear three or four times a day, after the meatus has been previously syringed out with simple water."—" In more severe cases it is also necessary to rub in tartar-emetic ointment behind the ear."—" If the patient be plethoric and accustomed to high living, and the discharge from the ear very abundant, he should be put on a spare diet, and be well purged several times a week with saline purgatives."

In these directions, with trifling variations, are comprised all the most important features of the treatment adopted by Dr. Kramer in the whole of the diseases of the external ear; the form of ointment, he remaks is not desirable—" the glandular structure does not bear well greasy applications." —" Blisters, when any derivation is necessary, are far too feeble in their action, and at the most do not affect what is accomplished by tartar-emetic ointment. Leeches are quite superfluous, and for bleeding and a general antiphlogistic treatment, in catarrhal inflammation of the ear, there is not the least indication."

In a case immediately following, however illustrative, we presume of inflammation of the glandular structure of the meatus under which heading it appears—" Four leeches were applied over the mastoid process, an emetic and warm almond oil dropped into the meatus"—improving the patient's hearing, and a weak solution of acetate of lead, we are informed completed the cure.

Under the same head we find another case of a child presenting "a discharge of a strongly and offensively smelling purulent fluid mixed with streaks of blood from both ears," relieved and cured "by careful diet, a due regard to the evacuation of fæces, an injection of acetate of lead gr. iv., ad aquæ ℥ij. thrice daily during four weeks, and then substituted by pyroligneous acid ℈ij. ad aquæ ℥ij."

In cases of phlegmonous inflammation tending to suppuration, the same treatment as that which is universally adopted, wherever the inflammation may take place, is recommended—leeches, poultices, &c., with the addition of warm almond oil occasionally dropped into the ear. Dr. Kramer and his translator are rather at issue on one point—the author never observed earache without evidence of inflammation either of the meatus or of the membrana tympani, while Dr. Bennett is not prepared, and we think with reason, to deny the existence of a nervous otalgia. In polypi of the membrana tympani radical treatment rarely or never succeeds. In acute inflammation of that membrane—the chief diagnostic sign of which seems to be pain and febrile symptoms; the antiphlogistic treatment finds favour, and the acetate of lead is forbidden.

"If, however, inflammation has seized on the whole extent of the membrana tympani, if it be very painful and swollen, and the patient feverish, ten or more leeches should be put on around the ear, emollient poultices applied, warm almond oil dropped into the ear, and powerful saline purgatives given. Solution of acetate of lead is here quite improper; it only favours the thickening of the membrane."

"Should, however, a muco-purulent secretion have been already established, with or without the destruction of the membrana tympani, next to tartar emetic ointment, solution of acetate of lead, dropped into the ear, is the remedy from which we may expect the most powerful assistance." 152.

The perforation of the membrana tympani and the various instruments devised for that purpose are discussed; he prefers Hinly's punch, which removes a circular portion, or if the membrane be cartilaginous, an instrument with two cutting barbs invented by Deleau. This operation is only indicated when the deafness arises from the thickness or disease of the membrane, and that only—it being distinctly contra-indicated when there is co-existent alteration of structure in any other part:—thus speaking of the operation he says:

"When the membrana tympani is only slightly thickened, that is, without cartilaginous degeneration, it readily yields to the pressure of the instrument, and a hand whose sense of touch is delicate, distinctly perceives when the opposition of the membrane is overcome. A drop of blood flows from the small wound; and occasionally the patient feels languid, and disposed to faint. Should a puriform mucus flow from the opening, and a similar matter be remarked on the punch, the cavity of the tympanum is diseased, and the affected ear cannot be expected to derive any benefit from the operation. In this case, either the diagnosis has been incorrect, or the operation has been undertaken on false grounds." 161.

Under the head of "diseases of the middle ear," we do not meet with so many fine drawn distinctions—they are thus defined:

"Under the title of 'Diseases of the Internal Ear,' Saissy has included every disease of the ear, with the exception of those of the auricle, and of the meatus. This, however, is improper, as the diseases of the cavity of the tympanum, and of the Eustachian tube, not only arise quite independently of those of the labyrinth, but also require a very different, and even opposite, plan of treatment. For these reasons, and for the sake of affording a more convenient view of them, I include among diseases of the middle ear, those only which occur in the cavity of the tympanum and in the Eustachian tube, and which are accessible, at least to our means of diagnosis, duiing the life of the patient, if not to our curative efforts." 187.

"Inflammation of the mucous membrane of the Eustachian tube, and of the cavity of the tympanum, with its various terminations and sequelæ; together with inflammation of the cellular tissue, situated beneath each of these membranes, are the only diseases that can be distinctly recognised, and these, therefore, are alone inserted here."

"It would be perfectly useless, on mere theoretical grounds, to attempt to separate diseases of the Eustachian tube from those of the cavity of the tympanum; for they present no peculiar marks by which they can be distinguished, which from the intimate connexion between the Eustachian tube and the cavity of the tympanum, must be the case. But if any such separation could be made, it would be void of any practical utility; for the diseases of both always require one and the same plan of treatment." 188.

The best mechanical means of investigating and treating diseases of the Eustachian tube and cavity of the tympanum are described at length, from which we learn that Dr. Kramer prefers, in catheterism of the tube, the inflexible silver catheter, with a well rounded extremity, six inches long, varying in calibre from a crow quill to that of a large goose quill, and curved only to the distance of five lines from the further extremity, exactly at the angle of 144°, so as to correspond with the lateral situation of the mouth of the Eustachian tube. He objects decidedly to elastic catheters, which Deleau and other aurists have recommended. He introduces the instrument through the middle meatus, and has practised the operation in "many hundred patients, on the whole many thousand times," proving that, with a fair degree of manual dexterity and knowledge, the difficulties are not great.

"If the air-douche is now to be made use of for the investigation of the middle ear, the patient sits close to a table, on which he leans the elbow of the affected side, and with the hand of the same side, lays hold of the tube of the air-press, which must previously have been charged. The operator is then to introduce the metallic tip of the tube into the funnel-shaped extremity of the catheter, and place his own ear close to that of the patient, which is to be examined; and having opened the cock of the machine, he listens to the noise which the condensed air makes in rushing into the ear of the patient. It would be quite out of place here, to describe the modifications of the sound thus heard, as the results of careful observation on this subject can be detailed, only along with the diagnosis of particular diseases of the middle and internal ear. I may simply mention here, that if the Eustachian tube and the cavity of the tympanum are completely free and open, the air rushes in unrestrained, and strikes with an audible shock against the membrana tympani. When the first shock of this forcible stream of air is over, or if it has not been so powerful, there is heard, from the continued stream of air rushing into the ear, a blowing and rustling, which appear to issue from the external meatus, and fill the whole ear of the patient. All deviations from this noise (the peculiarity of which can be rendered clear and comprehensible, only by repeated observation) are morbid, and afford very certain conclusions as to the particular diseased changes in the organic and functional condition of the ear. Should no air at all pass up to the membrana tympani, a catgut bougie should be introduced into the Eustachian tube, which we should try to push up to the membrana tympani." 201.

At page 195 the author says, speaking of aqueous injections, " they are attended with great difficulties and defects, of which I have been abundantly convinced by repeated experience." Then follows, under five heads, an enumeration of these difficulties, defects, and even dangers. Some 30 pages further we find the following passage.

"Till within the last few years, I also have made use of the water-douche with the greatest advantage and success, as will be evident from numerous cases to be detailed. And I must still declare my opinion that it is extremely useful in this affection, and cannot at all concur in the exaggerated and imaginary objections which Deleau makes to it. Nor is it attended by danger; on the contrary, it is advantageous to dissolve a little common salt in the water which is to be injected." 213.

It is difficult to discover what may be the Doctor's real opinions, the two here quoted being so completely at variance with each other; we strongly recommend *consistency* of opinion in the same book at least. In a subsequent paragraph the air-douche is given the preference from its facility, cleanliness, &c.

The middle ear we find liable to inflammation of its lining mucous membrane, the most common consequence being an accumulation of thickened mucus plugging up the Eustachian tube and leading to deafness with thickening of the membrane; when carried to great excess, either obstruction or obliteration of the canal may ensue. For the former disease Dr. Kramer relies upon the forcing a volume of condensed air to the membrane from the opening of the Eustachian tube, or a stream of water by the same means, by which the mucus is dislodged, the parts cleansed, and the disease gradually diminished. In the increased degree of stricture, a catgut bougie is used to dilate, passing it through the catheter; for obliteration there is no remedy. If there be inflammation of the fauces and adjoining parts, that ought first to be treated—the middle ear being lined by a continuation of the same membranes.

With respect to the symptoms of these forms of disease, deafness more or less complete is the only one constant—the catheter and the stream of air passing through it, afford the only means of arriving at an accurate diagnosis.

We cannot however avoid calling in question the truth of Dr. Kramer's conclusion, in opposition to obvious analogy, when he says:

"Nor does the nature of the disease become in the least changed, in course of time; it is, and continues to be, an accumulation of mucus, however long it may exist. It never passes spontaneously into stricture or obliteration of the Eustachian tube, if no more acute inflammation again attack the mucous membrane. It is for this reason that I have separated stricture and obliteration of the Eustachian tube, from that condition which consists in its engorgement from mucus, and have considered the former as distinct, independent diseases, by which means their diagnosis is also rendered more clear." 206.

In this, which is called the catarrhal inflammation—

"The prognosis is altogether favourable; even when the disease has been neglected, and has become firmly rooted, from having lasted for years; a complete cure, or very material improvement, may be effected by submitting the patient to a proper plan of treatment." 207.

When the disease is confined to the ear, Dr. Kramer concludes that general treatment can produce little benefit. And indeed it is evident throughout that the treatment relied upon in these affections is nearly entirely topical, the efficacy depending upon the judgment exercised in the selection, and the dexterity of the application of, a very limited number of remedial agents. The following paragraph distinctly enforces, as a general principle, that—

"All remedies that act generally, and which are employed for the sake of carrying into effect the causal indication, should be rejected; for this reason also, that, in order to act on a very isolated organ, to which we have easy, immediate, and certain access, they must commence their action from a great distance, and place the whole organism (in other respects often perfectly healthy) under contribution, without obtaining, after all, more certain favourable results." 209.

The prognosis, when disease has extended to the filling up of the tube by engorgement or to stricture, is in every respect unfavourable, "by air I have never been able to effect any real dilatation of the Eustachian tube." The obliteration of the canal is a very rare disease, and Dr. K. has only met with it when both ears have been simultaneously affected. The inflammation of the cellular tissue and periosteum of this cavity, which Dr. K. defines as the true internal inflammation of the ear, acute and chronic, seems to be the only disease involving life and producing general disturbance. This is attended by febrile symptoms of a decided character, "acute pricking, burning, tearing, boring, and dragging pains, are felt at the bottom usually of only one ear." The disease extends to the mastoid process, extending even to the dura mater and brain, involving all the surrounding bony and soft parts, ending in delirium and death. Little seems to be known of the cause of so violent a disease—some doubt has existed whether it ever is a primary affection, and our author attacks with some asperity Abercrombie, for having apparently called it in question. Dr. Kramer gives his opinion very decisively of its primary character, but without furnishing any proof. Cold—or metastatic transference of the cutaneous inflammatory action in scarlatina, small-pox, &c. are described as causes for its appearance.

An energetic antiphlogistic treatment—general bleeding from the jugular —leeches in large number around the affected ear—calomel in large doses given alternately with full doses of purgative salts—mercurial ointment rubbed in about the ear, and when the cerebral affection admits of it, emollient poultices to the ear—the meatus filled with warm water or almond oil, is the treatment recommended. The mastoid process should be laid open whenever fluctuation is discovered.

Under the head "Diseases of the Internal Ear" are included diseases of the labyrinth; that is to say, of the vestibules, the semicircular canals, the cochlea, and the nervous expansion, enclosed within these cavities. For these we have intentionally left but little space, since their existence and treatment are alike hypothetical—in the present state of the art at least they are neither of much practical importance nor interest. From these spring what is termed "nervous deafness," which, when it really exists, and is not used merely to cloak ignorance by a name, is beyond the reach of art. The diagnostic signs are rather negative than positive—and consist chiefly in our means of ascertaining that the external and middle ear is *not* diseased, ergo—when loss of hearing, &c. occurs, the internal must be so.

The principal exciting cause is considered to be cold. All debilitating influences act in a very distinctly injurious manner, such as grief, care, &c. and violent concussions. Attention to the general health, and the passing a stream of ætherous vapour through the Eustachian tube—preference being given to acetous ether—is recommended for the erethitic form; when the deafness is decided to arise, on the contrary, from paralysis or torpor of the nerve, a more stimulant vapour is recommended. Inadequate as this treatment seems to be, several cases of success are reported.

In the chapter on ear-trumpets, Dr. K. says he considers the instruments best for all patients suffering from diseases of the ear, are those which are merely simple media for conducting and concentrating sounds, of which the conducting-tube of Mr. Dunber of Rathenow may serve as a model.

The last chapter is devoted to an investigation of how far deaf-dumbness may be curable; and although Dr. Kramer's conclusions are very disheartening, yet it must be evident that deafness may proceed from many causes, some of which can be removed, while others are altogether irremediable; so a careful examination into the causes, in each particular case of the infirmity, is of the utmost importance. It has been shewn that our means of investigation enable us very accurately to ascertain the state of the external and middle ear, and any deviation from health—and that they are very rarely beyond the reach of art—while the affections of the internal ear are infinitely more obscure, and form the only class, the majority of which are hopeless. Fortunately, in the diseases of the ear, the most dangerous and obscure are also the most rare.

It will be obvious that we consider Dr. Kramer's work, which is clearly and well-translated by Dr. Bennett, to be one of no small merit. It is, doubtless, like most German productions, somewhat wordy and diffuse—and might have been compressed into one half its present size, without the least disadvantage to author or reader. The cases are unnecessarily numerous, and all previous authorities on acoustic medicine and surgery are handled with a roughness and acerbity not very usual amongst continental writers, and more in accordance with the rival *artists* on this side of the channel. Dr. Kramer does not seem to be aware that, in his sweeping strictures on acoustic writers, he engenders some scepticism as to the proficiency of the art itself; since, although he may think himself infallible, the world will not give him credit for infallibility. "*Rien n'est si doux que ce qui est fort,*" said a talented writer; and we are not the more disposed to believe an author right, because he vehemently maintains that his neighbours and predecessors are wrong. In respect to the translator, as he has not professed to abridge his original, his sole duty was fidelity—and that duty we believe he has performed with ability. The work, with all its minuteness of detail and German amplification, will be very useful in this country, where aural science is at an exceedingly low ebb, as compared with ophthalmic and dental practice.

